# A systematic autopsy survey of human infant bridging veins

**DOI:** 10.1007/s00414-017-1714-3

**Published:** 2017-10-26

**Authors:** Emma C. Cheshire, Roger D. G. Malcomson, Peng Sun, Evgeny M. Mirkes, Jasmin M. Amoroso, Guy N. Rutty

**Affiliations:** 1 East Midlands Forensic Pathology Unit, Robert Kilpatrick Building, Level 3 Leicester Royal Infirmary, University of Leicester, Leicester, LE2 7LX UK; 2 Histopathology Department, Leicester Royal Infirmary, Infirmary Close, Leicester, LE1 5WW UK; 3 Mathematics Department, University of Leicester, University Road, Leicester, LE1 7RH UK

**Keywords:** Bridging vein, Head injury, Child, Abuse, Subdural haemorrhage, Post-mortem

## Abstract

In the first years of life, subdural haemorrhage (SDH) within the cranial cavity can occur through accidental and non-accidental mechanisms as well as from birth-related injury. This type of bleeding is the most common finding in victims of abusive head trauma (AHT). Historically, the most frequent cause of SDHs in infancy is suggested to be traumatic damage to bridging veins traversing from the brain to the dural membrane. However, several alternative hypotheses have been suggested for the cause and origin of subdural bleeding. It has also been suggested by some that bridging veins are too large to rupture through the forces associated with AHT. To date, there have been no systematic anatomical studies on infant bridging veins. During 43 neonatal, infant and young child post-mortem examinations, we have mapped the locations and numbers of bridging veins onto a 3D model of the surface of a representative infant brain. We have also recorded the in situ diameter of 79 bridging veins from two neonatal, one infant and two young children at post-mortem examination. Large numbers of veins, both distant from and directly entering the dural venous sinuses, were discovered travelling between the brain and dural membrane, with the mean number of veins per brain being 54.1 and the largest number recorded as 94. The mean diameter of the bridging veins was 0.93 mm, with measurements ranging from 0.05 to 3.07 mm. These data demonstrate that some veins are extremely small and subjectively, and they appear to be delicate. Characterisation of infant bridging veins will contribute to the current understanding of potential vascular sources of subdural bleeding and could also be used to further develop computational models of infant head injury.

## Introduction

In early childhood, subdural haemorrhage (SDH) can occur through accidental and non-accidental mechanisms [1–4] as well as from birth-related trauma [5]. One of the proposed non-accidental mechanisms of injury involves a baby being subjected to repetitive rotational and acceleration/deceleration forces within the head due to violent shaking, with or without an additional impact force. The term abusive head trauma (AHT) is often used to include a pattern of injuries that are thought to occur through the inertial forces associated with a shaking event. Of these injuries, SDH is the most commonly observed feature and has been demonstrated in up to 92% of AHT cases [6].

The origin of infantile SDH has been debated throughout the scientific and medical literature [7–9]. Historically, damage to the bridging veins has been the most commonly suggested source of subdural bleeding in AHT [10–12]. However, previous experimental research on head injuries has centred around animal and biomechanical models [13, 14]. These studies have been used to estimate the amount of force required to tear bridging veins (which some authors state are large blood vessels [15, 16]) to magnitudes that would be far greater than that induced by inertial forces, such as those that can be produced by vigorous shaking. Direct evidence of ruptured bridging veins is also limited, demonstrated in only a few imaging studies by the extravasation of contrast agent [17] and during autopsy examination [18].

There are several detailed descriptions from neuroimaging and cadaver dissection studies on the locations, size and anatomical characteristics of adult bridging veins [19–21]. However, there is scanty published research on infant bridging veins, which would be expected to be proportionally smaller than adult vessels. The most comprehensive studies on the anatomical locations of adult bridging veins can be found in neurosurgical books and papers as it is crucial for neurosurgeons to be aware of these veins during various surgical approaches, as damage to a bridging vein can result in post-operative brain damage [22–24].

In this study, we map the number and anatomical locations of bridging veins from 43 human neonatal, infant and young child post-mortem examinations onto a 3D model of the surface of a representative infant brain. We also describe our observations of the drainage routes of these vessels, either directly into the dural venous sinuses or indirectly through venous pathways within the dural membrane. The mean diameter of 79 bridging veins from five additional cases within these age groups is also reported.

## Materials and methods

### Case selection

Forty eight neonatal, infant and early childhood (up to 2 years of age) autopsies undertaken at Leicester Royal Infirmary between March 2014 and January 2017 as part of a regional paediatric autopsy service were included in this study (Table 1). Corrected gestational ages were recorded for neonates within the first week of life. The first 43 cases of the series were used for the 3D mapping of vein locations and total counts of vein numbers. Five subsequent cases were used for in situ measurement of bridging vein diameters (a 1-day-old male and a 1-day-old female, a 29-day-old female, an 18-month-old male and a 2-year-old male). For all cases where in situ measurements were made of bridging veins, appropriate parental consent was given. Retrospective ethical approval was also obtained for the use of archived autopsy photographs of paediatric brains and bridging veins for research purposes (NRES 14/EM/0169).

**Table 1:** Age, sex, cause of death, total number of bridging veins and presence of SDH in the case series

Case	Age	Sex	Cause of death/associated features	No. of veins	SDH
Neonatal group (˂ 28 days)					
1	36 + 3 weeks GA	M	HIE	53	N
2	1 day	M	Birth trauma	n/a	Y
3	1 day	F	Pulmonary haemorrhage	n/a	N
4	1 day	F	Birth trauma, subgaleal haemorrhage	67	Y
5	1 day	F	Perinatal asphyxiation and head injury	30	Y
6	1 day	M	Perinatal head trauma	45	Y
7	3 days	M	HIE, perinatal asphyxiation, uteroplacental insufficiency	71	N
8	3 days	M	HIE, uteroplacental insufficiency and ruptured vasa previa	45	N
9	3 days	F	Pulmonary haemorrhage; subtle congenital anomalies	51	N
10	3 days	M	Persistent pulmonary hypertension of the newborn, patent ductus arteriosus	51	N
11	6 days	M	Bowel perforation, perinatal head trauma	33	Y
12	8 days	F	Lung dysplasia	64	N
13	12 days	F	Positional asphyxia, co-sleeping	56	Y
14	17 days	M	HSV infection	54	N
15	24 days	M	Pulmonary haemorrhage	43	N
16	26 days	F	Unascertained, SUDI, co-sleeping	58	N
Infant group (4 weeks to 1 year)					
17	4 weeks	M	Pulmonary haemorrhage	66	Y
18	4 weeks	M	Unascertained, SUDI	62	Y
19	4 weeks	M	Ruptured cerebrovascular malformation	57	Y
20	4 weeks	F	Multiple organ failure, complex congenital heart disease	n/a	N
21	6 weeks	F	Unascertained, co-sleeping, possible positional asphyxiation	48	N
22	8 weeks	M	SIDS	57	N
23	8 weeks	M	Unascertained, SUDI, co-sleeping	51	N
24	9 weeks	F	SIDS	60	N
25	9 weeks	M	AHT	27	Y
26	9 weeks	M	External airway obstruction, co-sleeping	58	N
27	9 weeks	M	Unascertained, SUDI, co-sleeping	50	N
28	14 weeks	M	Overlaying, minor crush injury to head, co-sleeping	71	Y
29	15 weeks	M	SIDS	56	N
30	15 weeks	M	SIDS	63	N
31	16 weeks	F	Positional asphyxia, restrictive seating device	39	N
32	17 weeks	M	AHT	30	Y
33	21 weeks	M	SIDS	49	N
34	23 weeks	F	Unascertained, SUDI, co-sleeping	40	N
35	25 weeks	M	Unascertained, SUDI, co-sleeping	85	N
36	27 weeks	F	Dog attack, head injury	44	N
37	29 weeks	F	Smoke inhalation	54	N
38	31 weeks	F	AHT	34	Y
39	43 weeks	F	Unascertained, SUDI, co-sleeping	58	N
40	45 weeks	M	RSV bronchiolitis	69	N
Young children (≤ 3 years)					
41	13 months	F	Unascertained, possible external airway obstruction	94	N
42	14 months	M	HIE, cause unascertained	75	N
43	18 months	F	Unascertained, SUDI, prone sleeping, recurrent febrile convulsions	36	N
44	18 months	M	Unascertained, SUDIC	52	N
45	18 months	M	Aspiration of a foreign object and viral bronchiolitis (RSV and parainfluenza virus, type 4 positive)	n/a	N
46	20 months	F	Sharp force extracranial trauma	45	N
47	25 months	M	Bronchopneumonia, viral respiratory tract infection, recurrent febrile convulsions	n/a	N
48	29 months	M	Cystic encephalomalacia and epilepsy	75	N

*HIE* hypoxic-ischaemic encephalopathy, *HSV* herpes simplex virus, *SUDI* sudden unexpected death in infancy, SUDIC sudden unexpected death in childhood, *SIDS* sudden infant death syndrome, *RSV* respiratory syncytial virus, *n/a* no bridging vein count recorded as case consented for measurement of vessels

Included in the series were three AHT cases and five perinatal head trauma cases (Table 1).

#### Abusive head trauma cases

Abusive head trauma cases were classified as such using the following criteria: (1) extra-axial haemorrhage (subdural and/or subarachnoid), (2) injury to the brain (hypoxic-ischaemic injury, parenchymal injury, diffuse axonal injury, cerebral oedema), (3) retinal haemorrhages, (4) other injuries consistent with shaking/shaking with an impact (metaphyseal fractures, pattern bruises, spinal injury), (5) inadequate history to explain the observed injuries or confessed shaking and (6) defendant convicted of murder/manslaughter during legal proceedings.

Autopsy images of the subdural haemorrhages present in the cases below are available in our previous publication [25]. Case numbers given below relate to the current Table 1.

#### Case 25

Case 25 was a 9-week-old male with thin film SDH (Table 2). There were bruises to the head, face, chest, abdomen, back and right knee; old and fresh metaphyseal and corner fractures of the tibia, fibula and several ribs; subarachnoid haemorrhage over the surface of the brain; diffuse widespread ischaemia; focal and small amounts of βAPP deposition in the white matter of the cerebral hemisphere and brain stem structures; ischaemic changes in the spinal cord; severe retinal haemorrhages in the right eye and bilateral optic nerve sheath bleeding; and a single left eye retinal haemorrhage. The defendant was convicted of manslaughter.

**Table 2:** Description and locations of bleeding in cases with SDH, and mode of delivery for babies up to 4 weeks of age

Case number from Table 1	Age	Description of cranial SDH	Mode of delivery for babies ≤ 4 weeks
2	1 day	Thin patchy smear over entire convexity and within interhemispheric fissure. Thin film over supratentorial, middle fossa and cerebellar surfaces. Thicker film within posterior fossa	Normal vaginal delivery
4	1 day	Focal thin smear over the posterior parietal and occipital lobes. Focal thin film over the cerebellum	Caesarean section following failed ventouse delivery
5	1 day	Extensive, thick, space occupying, extending over entire convexities, within all fossae, both supra- and subtentorial. Thin film over cerebellar surface	Forceps delivery
6	1 day	Thin focal smear over right occipital lobe and underneath right occipital/temporal lobes. Thin film over surface of cerebellum and within the posterior fossa	Normal vaginal delivery
11	6 days	Thin smear covering right/left parietal, occipital and temporal lobes. Thin patchy supratentorial and within interhemispheric fissure. Extremely thin smear within middle fossa. Slightly thicker film within posterior fossa and over surface of cerebellum	Ventouse delivery
13	12 days	Thin smear of blood right/left occipital lobes	Forceps delivery
17	4 weeks	Extremely thin smear of focal blood over right/left occipital lobes. Small focal patches of old SDH under tentorium, occipital dura, supratentorial and within the interhemispheric fissure	Normal vaginal delivery
18	4 weeks	Trivial thin smear over left occipital lobe and surface of the cerebellum	Normal vaginal delivery
19	4 weeks	Intraventricular haemorrhage. Thin film of patchy SDH around right temporal pole, within interhemispheric fissure, over tentorium and under left temporal lobe. Thick SDH within the middle and posterior fossa	Normal vaginal delivery
25	9 weeks	Bilateral patchy thin film, thickest over sulci of brain with extremely thin bleeding over gyral convexities. Thicker blood over parietal/occipital lobes. SDH within interhemispheric fissure. Small smear of blood over surface of cerebellum	n/a
28	14 weeks	Patchy, extremely thin, over right convexity. Thin smears over right tentorium and falx	n/a
32	17 weeks	Bilateral patchy thin film, thickest over sulci of brain with extremely thin bleeding over gyral convexities. Thicker blood over frontal lobes and left parietal lobe. SDH in interhemispheric fissure and all cranial fossae	n/a
38	31 weeks	Bilateral patchy thin film, thickest over sulci of brain with extremely thin bleeding over gyral convexities. Thicker density of blood over left parietal lobe. SDH within interhemispheric fissure and middle/posterior fossae	n/a

The term ‘thin smear’ was used to describe a transparent area of blood, a ‘thin film’ was an opaque collection of SDH which was not space occupying (≈ or ˂ 2-mm thick) and a ‘thick’ SDH was opaque and space occupying (˃ 2-mm thick)

*n/a* infant older than 4 weeks of age

#### Case 32

Case 32 was a 17-week-old male with thin film SDH (Table 2). There was scalp bruising on the occiput with underlying skull fracture and damage to the skull sutures; subdural haemorrhage along the length of the spinal cord; subarachnoid haemorrhage over the brain and within the spinal cord; generalised hypoxic-ischaemic damage to the brain and axonal damage demonstrated by βAPP deposition; focal axonal damage to the spinal nerve roots; and bilateral and severe retinal haemorrhages. The defendant admitted to two separate occasions of shaking and was convicted of manslaughter.

#### Case 38

Case 38 was a 31-week-old female with thin film SDH (Table 2). There was bruising over the vertex of the scalp; subarachnoid haemorrhage over the brain and spinal cord; SDH and extradural haemorrhage in the spinal cord; generalised hypoxic-ischaemic damage to the brain and axonal damage in ill-defined areas of the brain and in the spinal nerve roots; and extensive bilateral retinal haemorrhages. The defendant was convicted of manslaughter.

### Post-mortem visualisation of the bridging veins and subdural haemorrhage

**Fig. 1:**
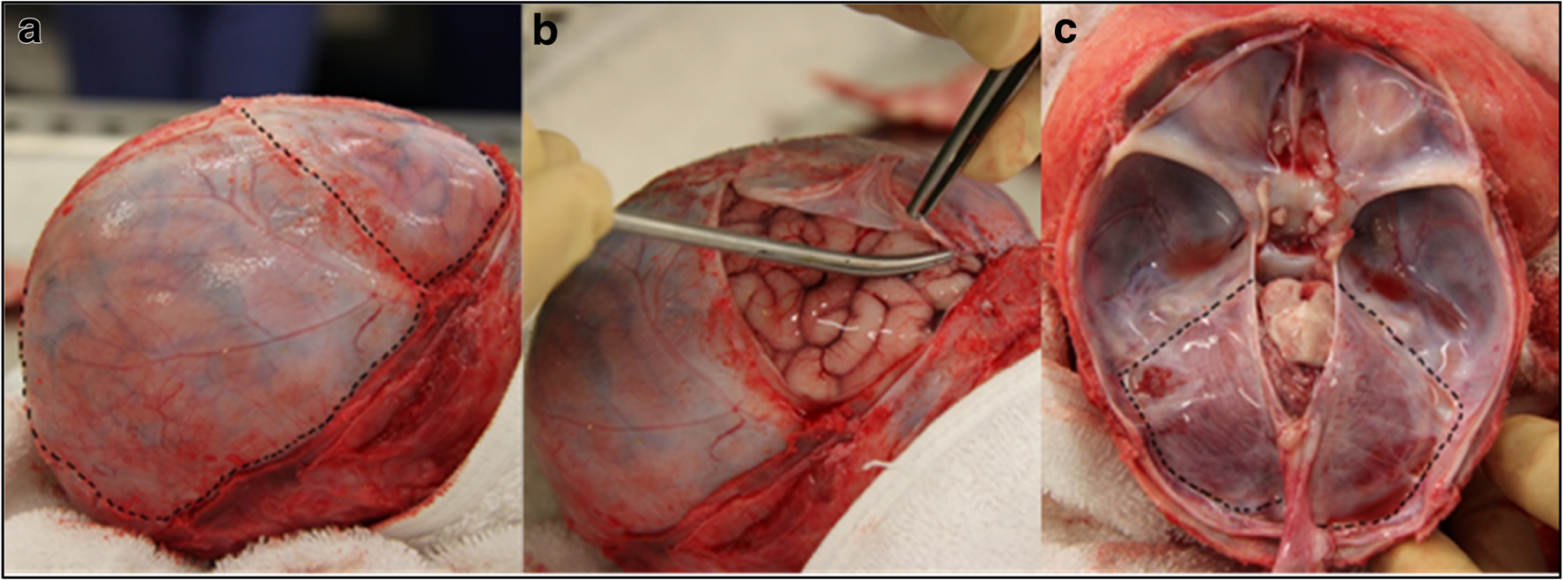
Incising and reflecting the dural membrane. **a** Incision lines along the right coronal and lambdoid sutures. **b** Reflection of the right frontal dura mater. **c** Incision lines on the tentorium

The calvarial bones were removed and the dura mater was optical cleared as previously described [25, 26]. Before reflection of the dura, any SDH seen through the membrane, overlying the cerebral convexities, was photographically documented. The dura was carefully incised according to the diagram (Fig. 1a). The dural flaps were then slowly reflected to enable photography of the bridging veins over the surface of the hemispheres (Fig. 1b). The brain was then gently tilted and manipulated to reveal bridging veins within the interhemispheric fissure, at the frontal and temporal poles, down the sides of the temporal lobe and on the undersurface of the brain, including veins associated with the tentorium. After a thorough examination of all the areas of the right hemisphere for bridging veins entering the dura, the right hemisphere was removed and a similar examination of the left side of the brain was conducted. The tentorium was then incised (Fig. 1c) and slowly reflected to reveal the bridging veins draining from the cerebellum. After the tentorium was lifted, the cerebellum was carefully manipulated to view any bridging veins remaining on the lateral and inferior surfaces. Vessels associated with the cranial nerves were not included. Subdural haemorrhage was further documented on reflection of the dura, after removal of each hemisphere, on reflection of the tentorium and after removal of the cerebellum. This allowed the presence of interhemispheric, supratentorial and subtentorial SDH to be recorded, as well as blood within the cranial fossae.

### Magnetic resonance imaging of an infant brain

For the purposes of creating a 3D model of an infant brain, magnetic resonance imaging (MRI) scans were undertaken on a brain from an infant with an age (11 weeks) close to the median of the case series, with specific parental consent. The brain was removed as part of the standard autopsy procedure and was placed in 20% formalin for approximately 2 weeks before scanning to allow for adequate fixation of the tissues. The brain was suspended in a specifically designed plastic container with the aid of two plastic rods to ensure that the organ was surrounded by fluid. This facilitated subsequent separation of the brain from the walls of the container on the MRI scans. Both T1-weighted and T2-weighted spin echo sequences were used to image the brain.

### Segmentation of the brain using ITK-SNAP

Using the T2-weighted MRI images (as the edges of the brain appeared to be more distinct in this sequence), the two hemispheres and the cerebellum were digitally isolated from each other by a combination of semi-automatic and manual segmentation using an open-source multi-platform software application (ITK-SNAP, version 3.2.0) [27] which is designed to segment structures in 3D medical images. Each part of the brain (hemispheres and cerebellum) was initially semi-automatically segmented, by outlining a 3D point of interest and by applying an algorithm to this area based on pixel intensity and image edges. Further manual segmentation was then required due to similar pixel intensities in the areas of interest and because the algorithm was unable to detect two small sections on the lateral aspects of the hemispheres due to reduced quality of the MRI images in those areas. Each segmented area of the brain was given a numerical label. The 3D brain was exported from ITK-SNAP as a mesh stl file. The numerical labels enabled the three separate components of the brain to be recognised by Matlab, a numerical computing environment.

### Creation of a Matlab Interface for plotting bridging vein locations on a 3D infant brain model

A user interface was developed using a commercial software package (MATLAB 2015a, The MathWorks, Inc., Natick, MA, USA) to enable the post-mortem photographic data to be mapped onto the 3D infant brain model produced in ITK-SNAP. The interface was created so that the brain could be viewed as its three separate components (hemispheres and cerebellum). This enabled plotting and visualisation of data points that would otherwise be hidden if viewing the brain as an entire organ (i.e. the interhemispheric fissure and the tentorium). To allow for data point plotting, the interface could rotate both the whole 3D brain and the separate hemispheres/cerebellum and also had the ability to save and load separate cases and groups of cases. A heat map function was added using ‘knnsearch’ and ‘rangesearch’ algorithms. The number of bridging veins on each of the hemispheres and the cerebellum were also recorded by the interface, allowing for the average number of vessels to be calculated for each of the separate parts and also for the whole brain.

### Macrophotography and measurement of bridging veins

**Fig. 2:**
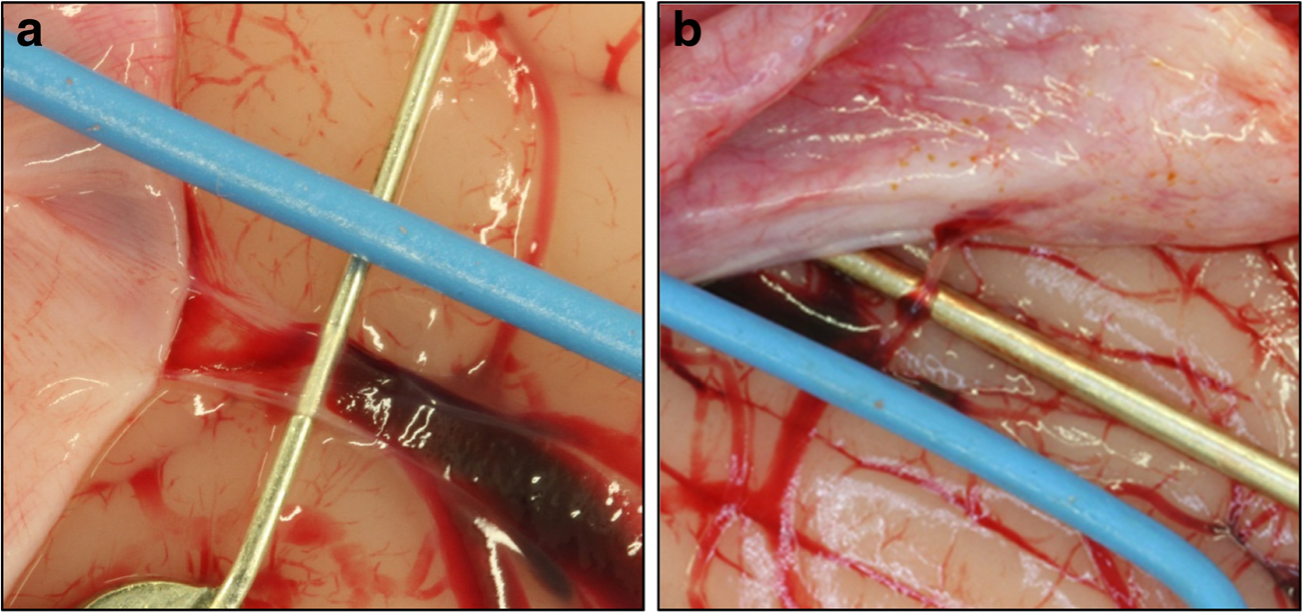
Digital macroscopic photographs of bridging veins with blue wire of known diameter. **a** Bridging vein on the left hemisphere in the frontal parasagittal region. **b** Bridging vein on the superior surface of the cerebellum

In five post-mortems, in situ measurements of the diameter of 79 bridging veins were recorded. The dura was reflected and easily accessible bridging veins (on the cerebral convexities including the parasagittal sections of the frontal and parietal lobes, within the interhemispheric fissure and on the superior surface of the cerebellum) were captured using a digital SLR camera. A blue plastic-coated wire of known diameter (0.22 mm) was placed adjacent to the each bridging vein and used as a coplanar scale reference (Fig. 2). Vessel diameters at the mid-point of the bridging section (the part of the vein which left the pia/arachnoid and joined either the dura mater or sinuses directly) were calculated using ImageJ image analysis software (National Institutes of Health, USA) from the pixel dimensions of the blue wire. Only singular vessels which were not obviously seen to branch within the bridging section of the vein were photographed for measuring purposes, due to the more easily defined edges of non-branched isolated blood vessels compared to branching vessels in close approximation.

## Results

### Post-mortem visualisation of the bridging veins and subdural haemorrhage

**Fig. 3:**
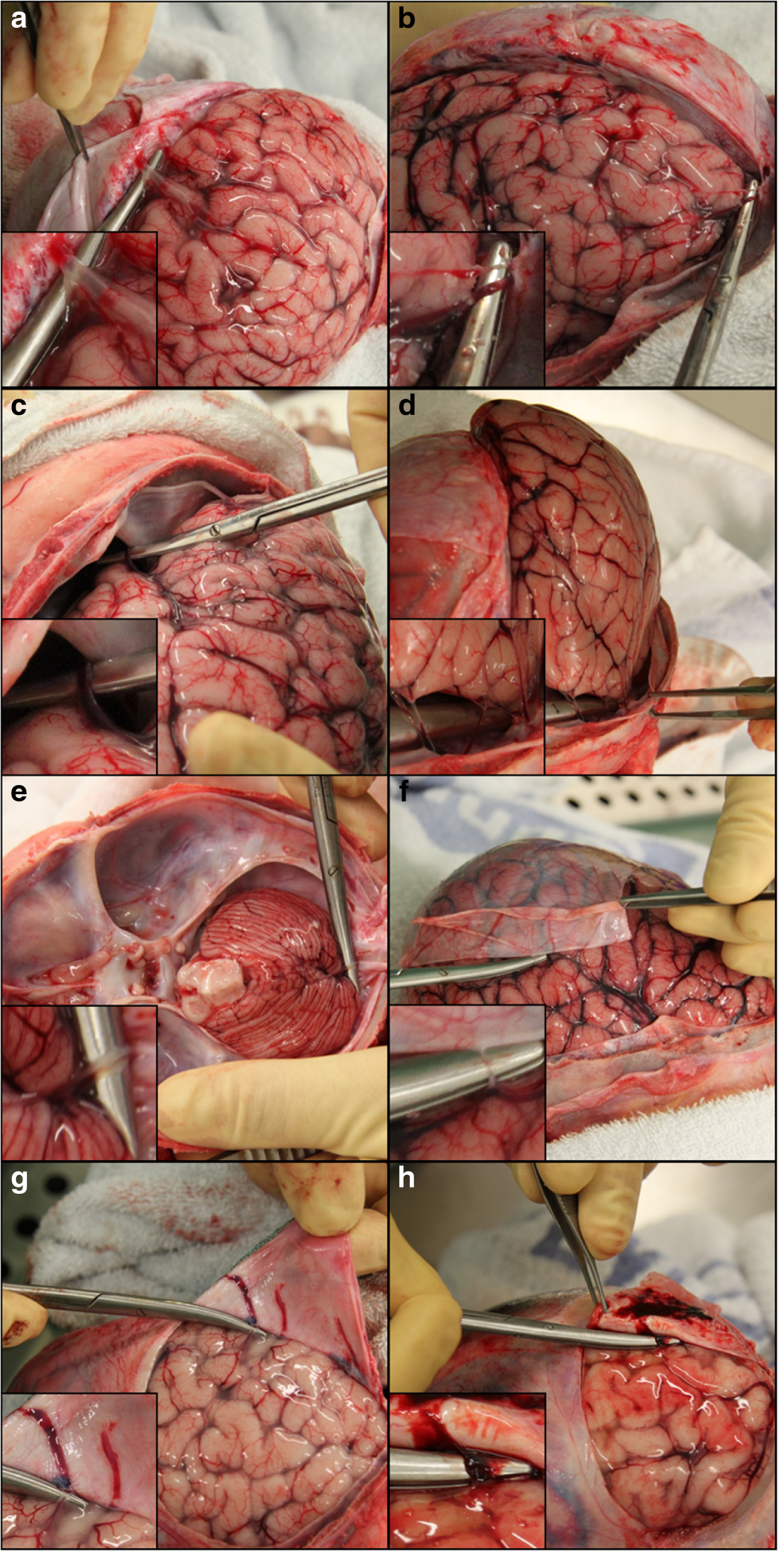
Reflection of the dura mater to reveal the bridging veins with ×2–4 magnification insets on the bottom left hand corner of each image. **a** Parasagittal bridging vein entering the SSS. **b** Bridging veins at the frontal pole. **c** Temporal pole. **d** Inferior aspect of the occipital lobe. **e** Bridging vein on the superior surface of the cerebellum. **f** Bridging vein distant from the sinuses, on the right parietal convexity. **g** Bridging veins running towards the SSS on the inner aspect of the dura. **h** AHT case with bridging vein engorged with blood and not blanching under slight pressure

**Fig. 4:**
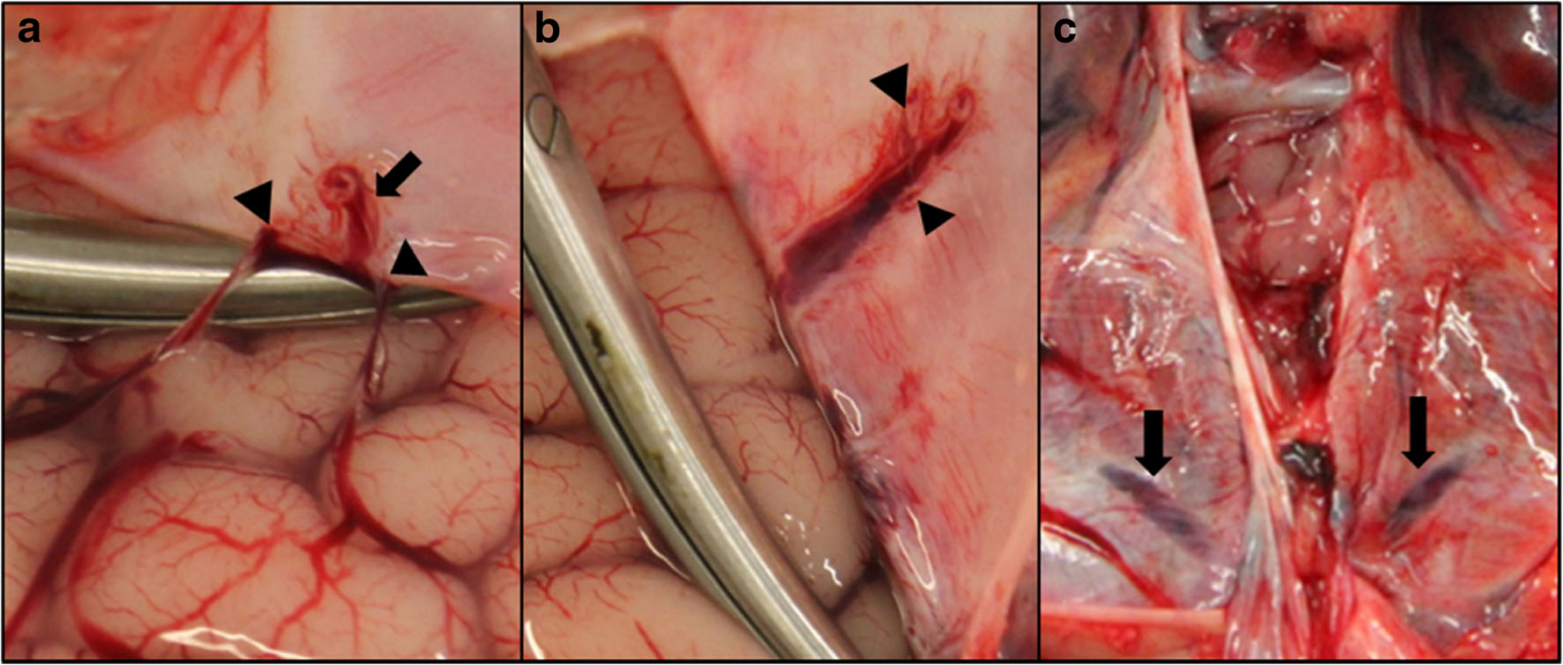
Parasagittal and tentorial sinuses. **a** Two bridging veins (*arrowheads*) draining the frontal lobe and entering a parasagittal sinus (*arrow*) approximately 3 cm distal to the SSS. **b** Parasagittal sinus from image **a** after cutting through the bridging veins and further reflection of the dura, *arrowheads* show the locations where the two bridging veins joined the sinus. **c** Tentorial sinuses (*arrows*) within the dura on both hemispheres of the cerebellum

We systematically located and photographically documented the macroscopically visible bridging veins in all 43 cases. The vessels were typically observed in the parasagittal region close to the superior sagittal sinus (SSS), including vessels which appeared to be directly entering the sinus (Fig. 3a). Further common bridging vein locations included at the frontal and temporal poles (Fig. 3b, c), the inferior aspect of the posterior temporal lobe/occipital lobe (Fig. 3d) and on the superior surface of the cerebellum (Fig. 3e). Although bridging veins were most often seen close to the venous sinuses, a few were also seen on the cerebral convexities distal to the sinuses and these vessels were often extremely small and could be missed when viewed with the naked eye (Fig. 3f). Veins that did not directly enter the more well-known, relatively large, dural venous sinuses, appeared to enter smaller venous channels on the inner surface of the dura (Fig. 3g), often in the parasagittal region, extending 2 to 3 cm from the midline (Fig. 4a, b) or within the tentorium (Fig. 4c). These venous channels had a flattened appearance, either within the inner aspect of the membrane itself or occasionally appearing to have a loose attachment to the membrane.

In the three AHT cases, the bridging veins tended to appear more obviously engorged with congested blood, particularly the larger vessels in the parasagittal region, and on application of slight pressure to the vessels, they did not blanch (Fig. 3h) (in a small study of a subset of 15 sequential non-head injured cases only 6 had blood vessels which did not blanch and of these cases only 1–3 veins in each case were observed to blanch under slight pressure).

In addition to the eight AHT and perinatal head injury cases, a further five neonates and infants also had SDH (Table 2). Of these five cases, only one infant was over 4 weeks of age, and this was a 14-week-old male with minor crush injury to the head due to overlaying. The three AHT cases had a similar pattern of bilateral patchy thin film SDH. No other cases demonstrated this particular pattern, but the appearance of SDHs in other cases ranged from small focal thin smears of blood to extensive space occupying haemorrhage.

### 3D mapping of the bridging veins

**Fig. 5:**
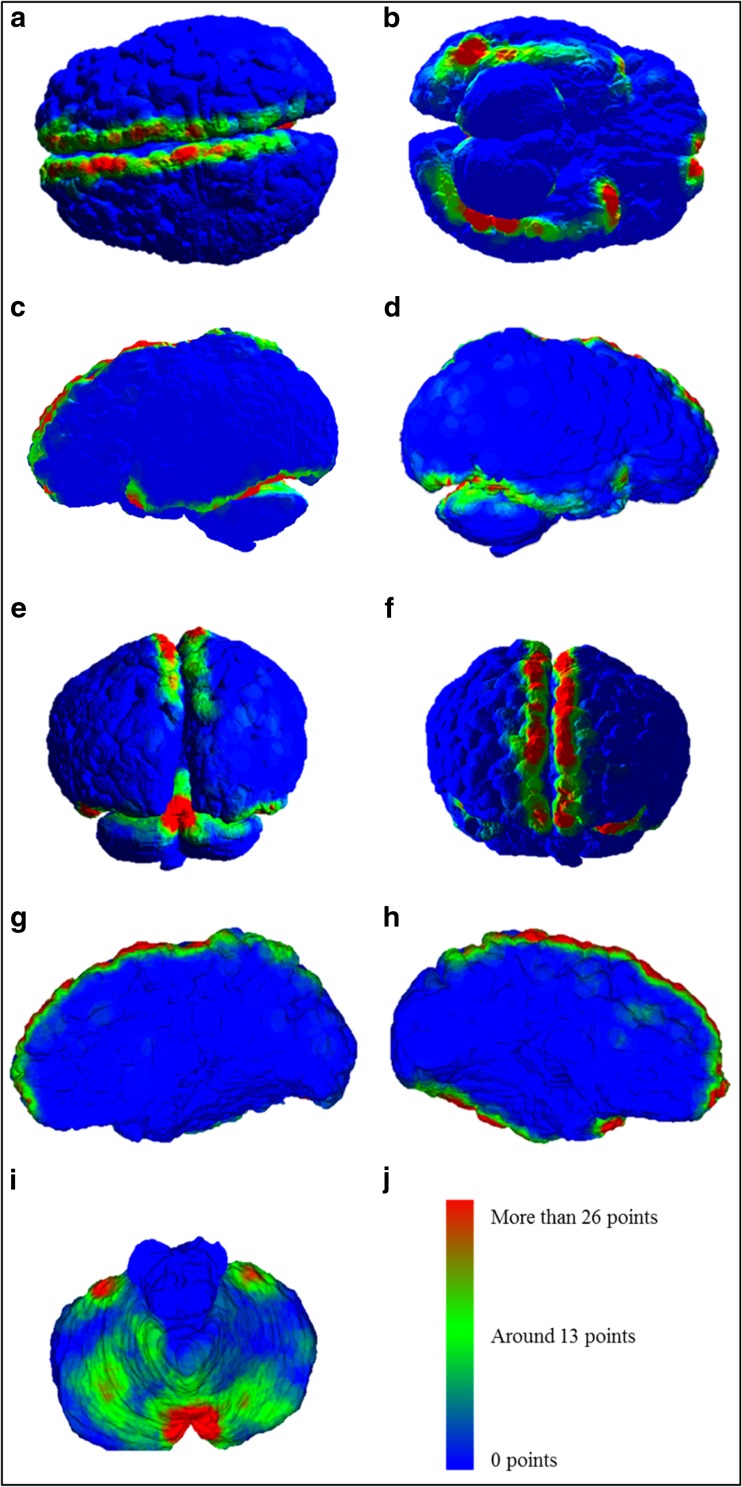
Heat map distribution of infant bridging veins. **a** Superior view showing interhemispheric fissure. **b** Inferior view showing vein distribution on the inferior temporal, occipital and frontal lobe. **c** Left lateral view. **d** Right lateral view. **e** Posterior view. **f** Anterior view showing veins near the frontal pole. **g** Interhemispheric fissure, right hemisphere. **h** Interhemispheric fissure, left hemisphere. **i** Superior view of the cerebellum. **j** Heat map intensity bar

Using the ‘knnsearch’ and ‘rangesearch’ algorithms to produce location density maps of the bridging veins, we identified large numbers of vessels near the frontal and parietal sections of the SSS, at the frontal and temporal poles, on the inferior aspect of the temporal and occipital lobes following close to the transverse sinus and around the superior vermis and the anterosuperior border of the cerebellar hemispheres (Fig. 5).

**Fig. 6:**
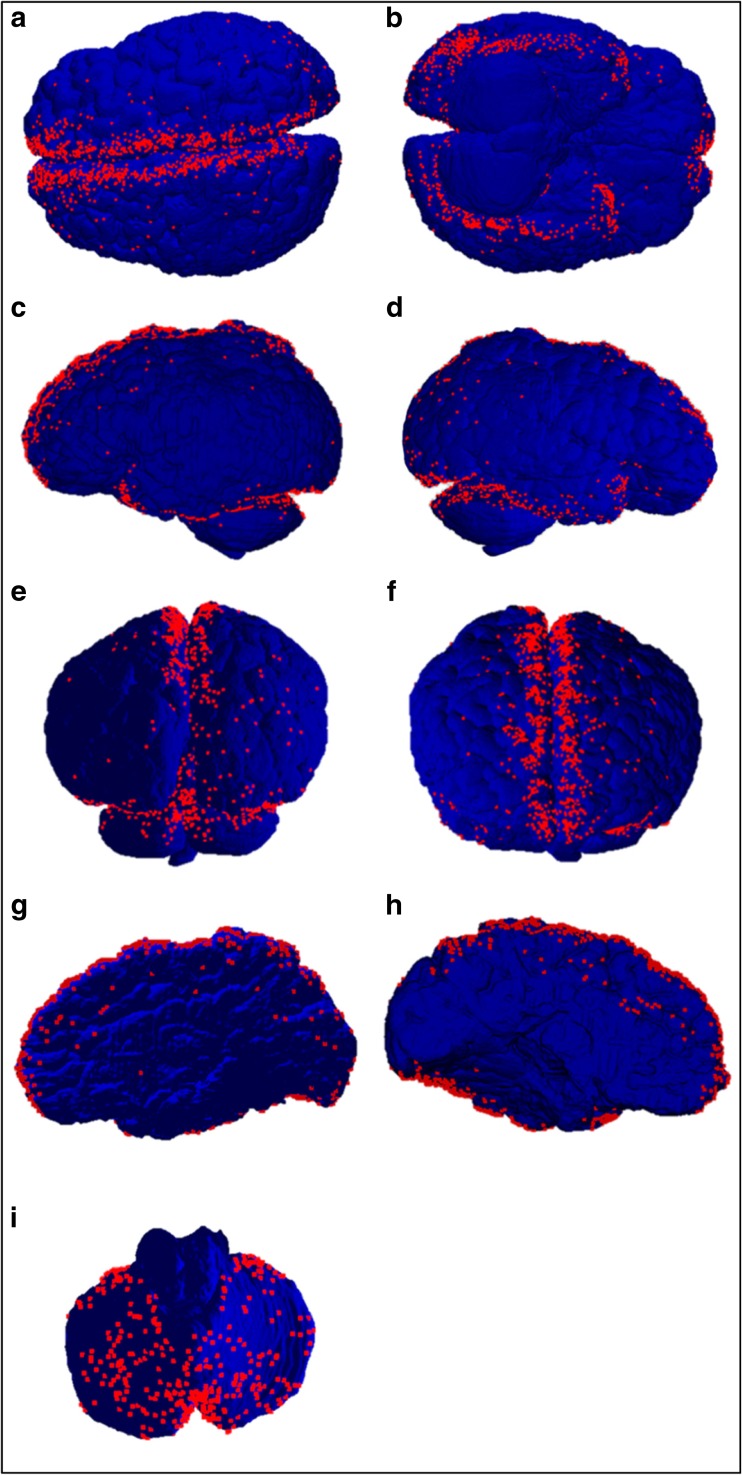
Dot scatter plots of infant bridging veins. **a** Superior view showing interhemispheric fissure. **b** Inferior view showing veins on the inferior temporal, occipital and frontal lobe. **c** Left lateral view. **d** Right lateral view. **e** Posterior view. **f** Anterior view showing veins near the frontal pole. **g** Interhemispheric fissure, right hemisphere. **h** Interhemispheric fissure, left hemisphere. **i** Superior view of the cerebellum

Although not demonstrated on the heat maps due to their smaller numbers and therefore smaller densities, when viewing the raw data points on the brain, bridging veins were also occasionally noted in locations situated further away from the sinuses during the post-mortem examinations (approximately 4%) (Fig. 6).

### Number of bridging veins

A total number of 2326 bridging veins were located on the 43 neonatal, infant and young child brains, with a mean of 54.1 per case. The total numbers of bridging veins in the series on the left and right hemispheres were 976 and 957, respectively. The mean numbers of bridging veins on the left and right hemispheres were 22.7 and 22.3, respectively, with no significant difference between the numbers on each hemisphere. The total number of bridging veins extending from the surface of the cerebellum identified in the series was 393. The mean number of bridging veins identified on the surface of the cerebellum was 9.1.

The five lowest counts for total bridging veins were seen in the three AHT cases included in the series (27, 30 and 34 veins) and two cases of perinatal head trauma (30 and 33 veins), born respectively by forceps and ventouse delivery methods (Tables 1 and 2). All five of these cases had SDH covering a significant amount of the cerebral surface and within the posterior fossa.

### Outer diameter of infant bridging veins

The mean diameter of the 79 bridging veins was 0.93 mm (range 0.05–3.07 mm). Due to the clustering and accessibility of bridging veins close to the SSS, most of the diameter measurements were taken from parasagittal vessels. In total, 37 bridging veins were recorded on the right hemisphere as opposed to 28 on the left hemisphere. Bridging veins in the interhemispheric fissure and cerebellum were also measured (Table 3).

**Table 3:** Mean diameter measurements of infant bridging veins related to location derived from five cases in the series

Bridging vein location	Number (*n*)	Mean diameter (mm)
Right frontal convexity	1	0.53
Left frontal convexity	4	0.94
Right frontal parasagittal	13	0.83
Left frontal parasagittal	10	0.92
Right parietal convexity	1	0.05
Left parietal convexity	0	n/a
Right parietal parasagittal	22	0.87
Left parietal parasagittal	14	1.38
Interhemispheric fissure	6	0.73
Cerebellum	8	0.74
Total	79	0.93

## Discussion

Post-mortem studies of the infant bridging veins are technically challenging due to their location below the dural membrane, as the infant dura is still attached to the calvarium at the sutures, rendering removal of the calvarium without damage to the bridging veins extremely difficult by conventional methods. When inspected at autopsy, bridging veins are fragile and easily disrupted by applying excessive stretching force during manipulation and under the force of gravity. Paediatric research is further complicated by the sensitivity of such studies, together with limited access to autopsy cases.

Light microscopy, X-ray, digital subtraction angiography and cadaveric anatomical dissections with vascular casting have previously been used to determine the diameter, number and anatomical locations of bridging veins [28–30]. Variation exists in the diameter measurements reported in these studies, with values ranging between 1 and 7 mm, except when using anatomical dissections where veins as small as 0.1 mm have been infrequently recorded [19]. In a radiological study, the diameters were shown to vary considerably between individuals but not in any one individual [28]. To our knowledge, no studies have specifically detailed the physical characteristics of infant bridging veins, with the exception of one study in which outer diameter measurements of between 0.14 and 0.72 mm were recorded for six vessels [31].

Owing to the delicate nature of the bridging veins, we were only able to measure the diameters of the larger bridging veins in the more accessible areas (i.e. the frontoparietal parasagittal regions and superior cerebellum). Due to the collapse of the blood vessels and lack of blood flow, post-mortem measurements can only be considered to represent approximations of the true in vivo vessel diameters. However, it is likely that the stated values for diameter measurements are over-estimations due to the flattened appearance of the veins from collapse and also due to the necessity of photographically documenting the veins lying against a very thin wire or probe to ascertain the bridging section for subsequent measurement of vein diameters. Although more bridging veins were measured from the right hemisphere than the left, it is possible that this is due to our standard method for opening the dura overlying the right hemisphere first, rather than reflecting anatomical asymmetry in these cases. Despite these limitations, the reported values were still considerably smaller than those documented in the majority of adult studies [29, 30] but broadly correlate with the limited measurements recorded by Morison for infant bridging veins [31].

There is considerable variation in the number of bridging veins recorded between studies in the literature [19, 20, 30], with average values ranging from 29 veins in one study [19] to only four or five in another study [20]. However, these studies often refer only to the larger veins draining directly into the SSS. Differences in reported numbers may also result from the various methods of investigation, cohort numbers and ages of the participants. A further area of study for bridging vein locations is underneath the tentorium, where vessels often enter the left and right transverse sinuses [32–35]. Neurosurgical papers also provide descriptions of bridging veins when determining the safety of several surgical approaches. However, these publications are usually focused on small, specific areas, often focused on the drainage of blood into a specific dural venous sinus [32, 36]. To our knowledge, this paper is the first systematic paediatric autopsy study to map all the bridging veins visible with the naked eye, in any one individual, over the entire surface of the brain. For this reason, our reported average number of infant bridging veins is considerably higher than values reported previously in studies in adult populations. It is probable that our average bridging vein count may nevertheless represent an underestimation owing to the technical difficulties of examining very small veins, documenting vessels located in cranial recesses with limited visibility and the necessity to incise the dura to gain access. However, we consider that our counts represent a best estimate of bridging vein numbers that could be identifiable by the naked eye under otherwise ordinary autopsy conditions. We have also demonstrated the considerable variation in number of bridging veins between individuals. Although our counts of bridging veins were lower in the AHT cases than the non-head injured cases, we cannot at this point determine whether this was due to the elastic recoil of small calibre broken veins, decreasing the likelihood of detection with the naked eye, or by high numbers of veins being obscured from view by the presence of SDH.

Our technique of using neurosurgical equipment and meticulous dissection methods have allowed us to demonstrate the anatomical locations of infant bridging veins, including vessels which ‘bridge’ to the dura distant from the sinuses and therefore demonstrating indirect routes of drainage into the larger dural venous sinuses. The locations of bridging veins recorded within this paper are comparable with previous publications [19, 21, 37], and although variations in the nomenclature used to describe these vessels exist in the published literature, they have previously been grouped as: a superior sagittal group, a sphenoidal group, a tentorial group and a falcine group, depending on which dural venous sinus they drain into [38].

Tentorial sinuses (sometimes described as meningeal veins or lakes) have been well described in the literature as constant venous channels [35, 37, 39]. Bridging veins from both the cerebellum and cerebrum may enter the tentorial sinuses which then drain into the transverse and straight sinuses including at the point of the transverse-sigmoid junction [40]. However, we observe that bridging veins also appeared to join venous channels either loosely attached to the dura or within the membrane itself, often in the parasagittal regions. Although not often alluded to in the literature, this indirect drainage route to the sinuses has been noted previously by some authors who describe ‘adhesions’ of the bridging veins to the dura [19] and venous sinuses (sometimes referred to as meningeal veins) [30, 38, 41] extending out by more than a centimetre from the SSS. In several more historical papers, as well as being described as either attached or within the dura, bridging veins are suggested to be free in the subdural space for up to 3 cm [42, 43], which one author describes as being particularly noticeable in the infant [42]. Through microscopic examination, the subdural space has been shown to be a potential space as the dura and arachnoid are continuous with one another [44]. A space is suggested to be created by the collection of fluid or air which cleaves through a weak cell layer of the dura [45] known as the dural border cell layer [46]. During our observations of the bridging veins, upon reflection of the dura, we create a subdural space; therefore, we cannot say how much of the free bridging sections of the veins were or were not within the dura. It is possible that the veins which appeared loosely attached to the dura may have occupied the dural border cell layer, disrupted by reflection of the membrane.

As bridging veins are the most commonly suggested source of infantile SDH, detailed descriptions of these blood vessels in the young are highly important for medical professionals involved in infantile head injury cases. We have shown that not all bridging veins are large calibre vessels and that some vessels are extremely small. They also appear to be delicate as they are easily disrupted on reflection of the dura.

As shown within this paper and others [20, 32, 37], the cerebral drainage system is extremely variable from one individual to the next. Whether this variation in vasculature could result in one infant being more susceptible to traumatic head injury and subdural bleeding than another cannot be answered here, but this point is worth future consideration, especially given the wide range of clinical outcomes seen in AHT cases. Anatomical information, including the data within this paper, may aid future understanding of the potential mechanisms which result in SDH and confirm whether or not bridging veins can be traumatically damaged by inertial/rotational forces to produce this type of bleeding. These data could also provide biomechanical engineers with the opportunity to create more detailed computational models of human infantile head trauma, accidental or otherwise.
